# Europium oxide nanoparticles (EONP) enhance the cryoprotective effects of chilled rabbit semen preservation via antioxidant and mitochondrial enhancement

**DOI:** 10.3389/fvets.2025.1753818

**Published:** 2026-01-27

**Authors:** Mohammed A. Alfattah

**Affiliations:** Department of Biology, College of Science, Jazan University, Jazan, Saudi Arabia

**Keywords:** chilled rabbit semen, europium oxide nanoparticles, mitochondria enzymes, nanozyme, semen quality

## Abstract

**Introduction:**

This study investigated the cryoprotective effects of europium oxide nanoparticles [nanozymes; Europium oxide nanoparticles (EONP)] on rabbit sperm quality during 72 h of chilled storage at 4 °C due to its robust antioxidant ability (mimicking enzymes) and mitochondrial enhancer.

**Methods:**

In this experiment, diluted semen samples were treated with different concentrations of EONP: 0 (EONP0), 25 (EONP25), 50 (EONP50), or 100 (EONP100) ng/ml. The treated samples were then stored at 4 °C for three days. Sperm quality attributes such as viability, motility, membrane integrity, and morphology were measured at 24, 48, and 72 h of storage. Oxidative/antioxidant activities and mitochondrial enzyme levels were also assessed after 72 h of storage.

**Results:**

The results show a quadratic relationship between EONP supplementation (at 25 or 50 ng/ml) and improved sperm motility, viability, and membrane integrity at all time points observed (*P* < 0.01). Additionally, a dose-dependent relationship was found between EONP levels and the concentrations of GPx, CAT, and SOD. In contrast, all groups supplemented with EONP showed a significant decrease in malondialdehyde (MDA), protein carbonyl (PC), hydrogen peroxide (H_2_O_2_), and nitric oxide (NO) levels compared to the extender without EONP (*P* < 0.001). The activities of succinate dehydrogenase (SD), malate dehydrogenase (MD), citrate synthase (CS), and ATP showed significant and quadratic improvements at EONP concentrations of 50 and 100 ng/ml compared to the 25 ng/ml concentration. Principal component analysis 1 (PCA1) was positively correlated with EONP supplementation, antioxidant markers, mitochondrial enzyme activities, and sperm quality parameters. Principal component analysis 2 (PCA2) showed a negative association between different EONP concentrations (0, 25, 50, and 100 ng/ml) and oxidative markers and sperm abnormalities after 72 h of preservation at 4 °C.

**Discussion:**

Nanozyme supplementation of rabbit semen extenders significantly improves sperm quality during 72-h chilled storage by increasing antioxidant competence, reducing oxidative stress (OS), and boosting mitochondrial enzyme activity. Our data suggest that europium oxide nanoparticles are a promising additive for chilled rabbit semen preservation, with implications for artificial insemination (AI) and rabbit breeding.

## 1 Introduction

Rabbits are a significant and often underestimated model in biomedical research (1, 2). Their rapid breeding, manageable size, and high prolificacy make them particularly valuable (3). Moreover, they offer unique advantages by effectively bridging the experimental gap between small rodents and larger animal models (4). Besides, rabbits have distinct physiological and anatomical characteristics that make them invaluable for studying a range of human diseases and for developing new diagnostics and therapeutics (5). The use of rabbits in reproductive technology research is of critical importance, especially concerning semen preservation (6, 7). They are invaluable for developing and testing semen extenders, assessing sperm quality parameters, and elucidating factors that influence sperm viability.

Artificial insemination (AI) plays a pivotal role in rabbit genetic improvement platforms, commonly using chilled semen on commercial farms (2, 8). While this practice typically yields satisfactory fertilization ratios, ongoing research aims to refine the most effective approaches for alleviating the deleterious effects of cold stress (1, 9). Low temperatures can negatively affect sperm function, structure, and fertilizing capacity, often resulting in diminished motility, compromised functionality, reduced viability, and mitochondrial dysfunction (1, 7, 10). This degradation is frequently associated with oxidative stress (OS), an imbalance between endogenous antioxidant competence and the generation of reactive oxygen species (ROS), which detrimentally affects cellular integrity and ultimately sperm function. A promising strategy to enable spermatozoa to resist cold-stress-induced free radicals involves incorporating chilled rabbit semen extender with natural antioxidants (1, 2).

Mitochondrial integrity is paramount for this vital cellular organelle, widely recognized as the powerhouse of the cell due to its critical role in producing energy for spermatozoa (11). This is because mitochondria are the primary sites of ATP synthesis via oxidative phosphorylation (12). Succinate dehydrogenase (SD), a key enzyme complex in this pathway, is essential not only for ATP production but also for sperm signaling, nucleotide synthesis, fatty acid metabolism and epigenetic regulation (12). Consequently, SD deficiency impairs oxidative phosphorylation, indicating insufficient mitochondrial energy and reduced ATP availability, which ultimately compromises sperm quality and can cause male infertility (11, 13). Malate dehydrogenase (MD) is an enzyme found in both the cytoplasm and mitochondria of sperm (2). It catalyzes the reversible interconversion of malate and oxaloacetate using NAD^+^/NADH, suggesting its importance in sperm metabolism (14). MD can impact sperm physiology and functional, ultimately contributing to successful fertilization (14). Citrate Synthase (CS) is the enzyme that catalyzes the first step of the Krebs cycle, combining acetyl-CoA (derived from glucose, fatty acids, or amino acids) with oxaloacetate to form citrate (15). This enzyme can initiate oxidative phosphorylation. Its ability to regulate the sperm penetration into the oocyte, and CS may indirectly influence other sperm functions, including the acrosome reaction, possibly via cAMP-dependent pathways (16). ATP is the essential fuel for sperm motility, capacitation, and the acrosome reaction (13). ATP serves as a substrate for adenylate cyclases to produce cAMP, a main second regulator in sperm signaling pathways that regulate sperm capacitation and the acrosome reaction (11, 17).

Rare earth elements (REEs) are integral to nearly every aspect of modern life due to their widespread use in energy, information technology, healthcare, environmental protection, agriculture, and defense (18). Among these REEs, europium (Eu) oxide refers to chemical compounds of europium and oxygen. The most common and stable form is europium oxide (Eu_2_O_3_), also known as europium sesquioxide or europium trioxide, which has different properties and applications (19, 20). Eu_2_O_3_ nanoparticles (EONP) show promise in diagnostics therapeutics, and imaging (21). EONP has been previously reported to possess immunomodulatory and anti-inflammatory effects (19). Eu-containing biomaterials exhibit both osteogenic and angiogenic properties, making them promising candidates for bone tissue engineering (21). However, it remains to be determined whether EONP promotes preservation of sperm quality by enhancing mitochondrial activity.

The aforementioned investigation recognized the anti-inflammatory, skin restoration, pro-angiogenic, and re-epithelialization actions of Eu_2_O_3_ nanoparticles (21). The EONP was utilized to enhance the antimicrobial capability of the methyl cellulose for use in antibacterial food packaging (20, 21). Previously, it has been indicated that the synthesized EONP from aqueous extracts of *Hyphaene thebaica* fruits were assessed for the antidiabetic potential through *in vivo* and *in vitro* bioassay (22).

Based on the biological therapeutic activity of EONP, the authors hypothesized that incorporating chilled rabbit semen with EONP can mimic the antioxidant enzymes and sperm mitochondrial bioenergetics enzymes, as well as counteract oxidative stress induced during the preservation process. Consequently, this investigation explores the impacts of EONP-fortified extenders on rabbit semen attributes over a 72-h storage period at 4 °C. This experiment focuses on oxidative homeostasis markers (oxidant/antioxidant signaling), sperm quality variables, and sperm mitochondrial bioenergetics enzymes. The study aims to provide valuable insights for optimizing short-term rabbit semen preservation strategies, ultimately contributing to advancements in mammalian assisted reproductive technologies.

## 2 Materials and methods

### 2.1 Ethics approval

The current study was conducted according to the guidelines of ARRIVE and approved by the Ethical Committee at the Faculty of Science, Jazan University, KSA.

### 2.2 Synthesis of europium oxide nanoparticles (EONP) nanozyme

Europium oxide (Eu_2_O_3_; CAS No. E808866), a product of Macklin (Pudong, Shanghai, China), was used in this study. The biosynthesis of Eu_2_O_3_ NPs was previously described in the article by Mohamed et al. (22). In a brief, the process began by combining 100 ml of an aqueous extract from dried fruit material with 4 g of europium nitrate hexahydrate (Eu(NO_3_)_3_·6H_2_O) precursor salt. This mixture was heated at 80 °C for one hour, then dried, and finally annealed in a glass tube furnace for two hours at 500 °C (Figure S1).

### 2.3 Animal and semen collection

This study involved 15 fertile New Zealand rabbit bucks, aged 12–14 months, with an average body weight of 3.3 ± 0.2 kg. Each buck was housed individually in a galvanized wire cage (35 × 70 × 50 cm) in a naturally ventilated room. The environmental conditions were maintained at an ambient temperature range of 16–25 °C and a standard photoperiod of 12 h of light daily.

To prepare the bucks for semen collection, they first received training with teaser does. Over two weeks, they were accustomed to using the artificial vagina (AV). Semen was then collected using an AV (maintained at 39–41 °C) mounted on a teaser doe. The gel plug was promptly removed post-collection, and the collected semen was transferred to a 37 °C water bath. All ejaculates were subsequently assessed for motility, concentration, and morphology via standard techniques (23). Ejaculates were included in the study only if they demonstrated a volume of 0.2 ml, ≥70% progressive motility, a sperm concentration of ≥200 × 10^6^ spermatozoa/ml, and ≥80% morphologically normal spermatozoa. Ejaculates falling short of any of these standards were excluded (7). A commercially formulated rabbit diet, providing 12.03% crude fiber, 15.8% crude protein, and 2,550 kcal/kg digestible energy, was fed to the animals, with fresh water available *ad libitum*.

### 2.4 Experimental groups and extender formulation

A total of 60 ejaculates were selected for this experiment, ensuring that a minimum of five samples originated from each buck. All collected semen samples initially experienced assessment based on pre-recognized standard measures; only samples satisfying these requirements were incorporated into the investigation. Recording this initial evaluation, the qualifying ejaculates were then pooled and diluted using a tris-citric acid-glucose (TCG) based diluent, achieving a final sperm concentration of 50 × 10^6^ spermatozoa ml^−1^. The TCG extender comprised of the following components: Tris (250.04 mM), streptomycin (75.00 IU), citric acid (79.76 mM), glucose (69.38 mM), and penicillin-G (166.20 IU). The osmolarity and pH of the dilution were carefully set to 299 mOsm kg^−1^ and 7.14, respectively (9).

The pooled extended semen was subsequently allocated to four treatment groups. The control group (EONP0) was created by further diluting the semen 1:10 with TCG extender. The other three groups incorporated Europium oxide nanoparticles (EONP) into the TCG extender at concentrations of 25 (EONP25), 50 (EONP50), and 100 (EONP100) ng ml^−1^. These extended semen treatments were then stored at 4 °C for 72 h. To monitor sperm quality over time, motility, viability, abnormal morphology, and plasma membrane integrity (PMI) were assessed at 24, 48, and 72 h of storage. At the conclusion of the 72-h storage, mitochondrial bioenergetics enzymes within sperm cells, as well as oxidative stress and antioxidant indicators in the semen were also determined.

### 2.5 Evaluation of sperm motility

We assessed sperm motility subjectively using a phase-contrast microscope (Olympus BX20, Tokyo, Japan) at 400 × magnification. The microscope was equipped with a heated stage maintained at 37 °C. For each assessment, a 10 μl aliquot of semen was placed on a pre-warmed (37 °C) microscope slide, covered with a coverslip, and checked directly (9).

### 2.6 Assessment of sperm abnormal morphology and viability

Sperm viability and abnormal morphology were assessed by preparing thin, uniform smears. Initially, a 10 μl aliquot of semen was mixed with 1% nigrosin-eosin stain (Sigma-Aldrich, St. Louis, MO, USA) on a pre-warmed glass slide. After air-drying, the smears were checked at 1,000 × magnification under oil immersion using a phase-contrast microscope (Olympus Corporation, Hachioji, Tokyo, Japan). Live spermatozoa were identified by their unstained heads, while dead or partially stained heads indicated dead spermatozoa. Using the same microscope, we assessed sperm morphology by recording the percentages of abnormal sperm cells. This included those with abnormal heads (microcephalic, pear-shaped, round, short, loose, or double) and abnormal tails (coiled, broken, terminally coiled, or double), all evaluated according to established protocols.

### 2.7 Measurement of sperm membrane integrity

To assess plasma membrane integrity, a 25 μl aliquot of extended semen (6 samples from each group) was incubated in 475 μl of hypo-osmotic solution (100 mOsm kg^−1^) at 35 °C for 15 min (10). Following the osmotic challenge, a wet mount was prepared, and approximately 200–300 spermatozoa were examined at 400 × magnification under a bright-field microscope. Sperm cells were classified based on the degree of tail curling, indicating intact or damaged plasma membranes. Subsequently, the percentage of spermatozoa with intact membranes was calculated.

### 2.8 Measurement of antioxidant status

After 72 h of storage, extended semen samples (*n* = 6, in each group) were centrifuged at 6,000 rpm for 10 minutes at 4 °C. The resulting supernatant was collected to measure the activities of catalase (CAT, CA2517), glutathione peroxidase (GPx, GP2524) and superoxide dismutase (SOD, SD2521). These enzymes were evaluated using commercially available kits based on the colorimetric method (Biodiagnostic, Giza, Egypt), following the manufacturer's procedures. Absorbance for these assays was determined at 505 nm using a spectrophotometer (Spectro UV-Vis Auto UV-2602, USA). The levels of malondialdehyde (MDA) (MyBioSource, Catalog No. MBS739495), protein carbonyl (PC) (MyBioSource, Catalog No. MBS1601647), nitric oxide (NO) (MyBioSource, Catalog No. MBS2540419), and hydrogen peroxide (H_2_O_2_) (MyBioSource, Catalog No. MBS822356) in the extended semen were determined using commercially available kits based on colorimetric methods (MyBioSource, San Diego, USA). All tests were conducted according to the respective kit protocols, and the measurements were taken using a spectrophotometer (USA, Spectro UV-Vis Auto UV-2602).

### 2.9 Assessment of mitochondria bioenergetics enzymes in sperm

Malate dehydrogenase (MD) succinate dehydrogenase (SD) levels were established utilizing ELISA kits from Nanjing Jiancheng Bioengineering Institute (Jiangsu, China). We followed the methods outlined in Zhu et al. (24). After 72 h of storage at 4 °C, the treated semen samples were centrifuged at 1,200 g for 15 min at the same temperature. Next, the sperm specimens were lysed ultrasonically using a 20 kHz, 750 W sonicator at 40% power for 5 cycles of 3 s on and 5 s off. The lysed samples were then centrifuged at 10,000 rpm for 10 min at 4 °C. The resulting supernatants were transferred to a 96-well plate to analyze SD and MD activities. A microplate reader was used for this analysis, with readings taken at 600 nm for SD and 340 nm for MD. The activities of both MD and SD were expressed as milliunits per milligram of protein (mU/mg protein).

Furthermore, the levels of citrate synthase (CS) in rabbit sperm were evaluated using a colorimetric detection system (MyBioSource, Catalog No. MBS722913). Adenosine triphosphate (ATP) levels in sperm were assessed using a colorimetric quantitative competitive ELISA Kit (Abbexa, USA). The kit has a test range of 1.56 ng/ml−100 ng/ml. Following the manufacturer's protocol, the optical density (OD) was measured spectrophotometrically at 450 nm in a microplate reader (25)

### 2.10 Statistical analysis

After entering data into Microsoft Excel 365, statistical analysis was performed using IBM SPSS Statistics version 25. To examine treatment-related trends, orthogonal contrasts (linear and quadratic effects) were conducted within a one-way ANOVA on all data. Graphs for data visualization were generated using GraphPad Prism version 9. A *p*-value of less than 0.05 was considered statistically significant. All results are presented as the mean ± SEM (standard error of the mean). PC-biplot and heatmap were created using the ggplot2 and RcolorBrewer packages in R software.

## 3 Results

### 3.1 Impacts on semen quality at 24 h

**Figure 1:**
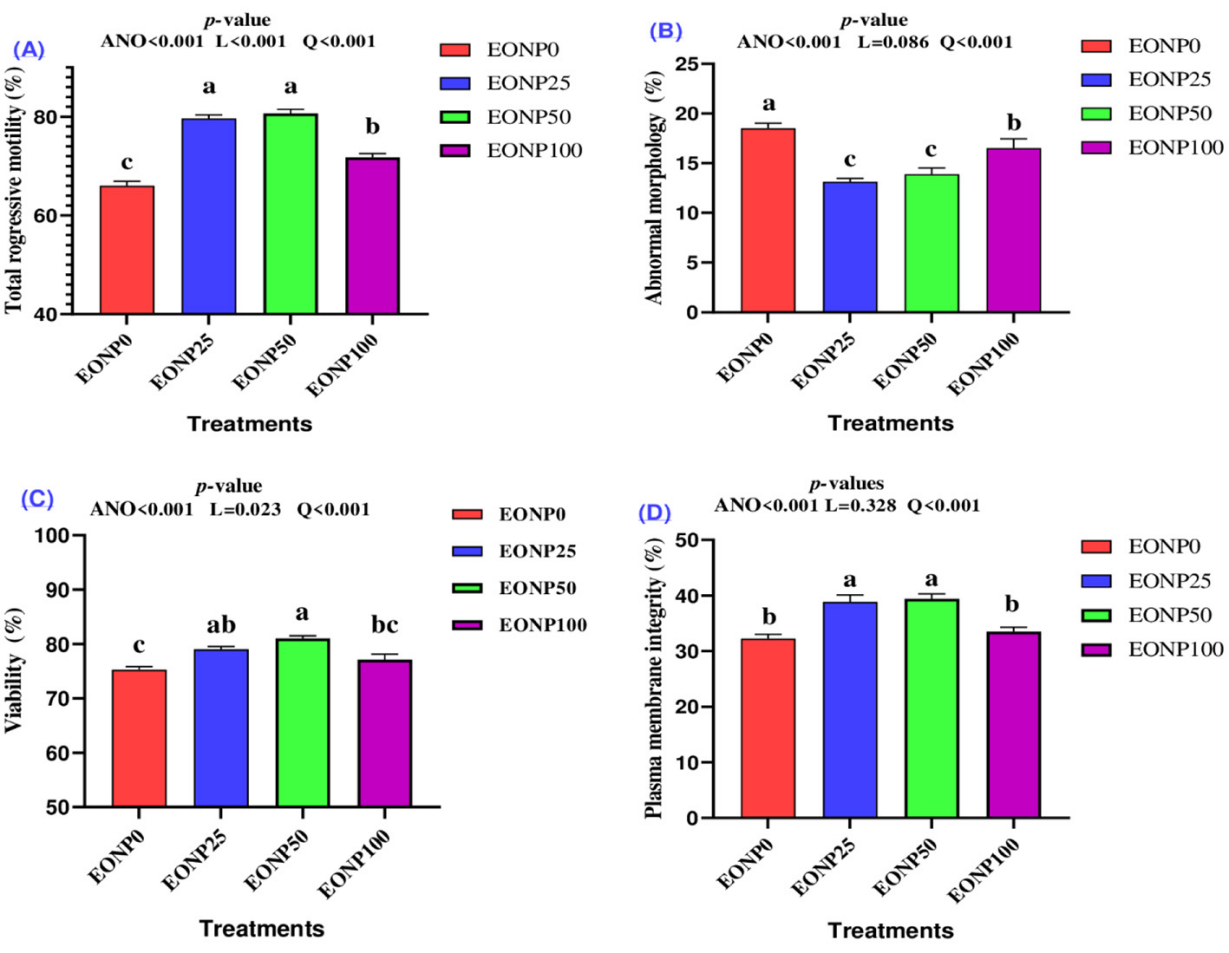
Outcomes of different concentrations of europium oxide nanoparticles (EONP; 0, 25, 50, and 100 ng/ml of extender**)** on chilled rabbit semen quality designated as EONP0, EONP25, EONP50, and EONP100, respectively. Treated semen was stored at 4 °C for 24 h for assesing total progressive motility **(A)**, abnormal morphology **(B)**, viability **(C)**, and plasma membrane integrity **(D)**. Bars displaying discrete superscript letters (^a, b, c^) suggest statistically significant differences (*P* < 0.05) among the treatment groups.

Figure 1 presents data on how varying levels of EONP affect total progressive motility, viability, abnormal morphology, and plasma membrane integrity (PMI) in chilled rabbit semen following 24 h of storage at 4 °C. Adding EONP at 25 or 50 ng/ml to the rabbit extender significantly improved total progressive motility in a quadratic manner compared to both the EONP0 and EONP100 groups (*P* < 0.001; Figure 1A). Additionally, the EONP100 group showed greater total progressive motility than the EONP0 group (*P* < 0.001). A quadratic decrease effect on sperm abnormal morphology was observed with EONP-enriched rabbit extender, with the EONP25 and EONP50 groups showing the lowest abnormal morphology values (*P* < 0.001; Figure 1B). Sperm viability was quadratically greater in chilled semen when supplemented with either 25 or 50 ng/ml of EONP (Figure 1C). Interestingly, there was no significant difference in sperm viability between the EONP25 and EONP100 groups (*P* > 0.05). Furthermore, adding 25 or 50 ng/ml of EONP significantly enhanced the PMI of chilled rabbit semen (*P* < 0.01; quadratic effect; Figure 1D). However, the highest dose of EONP had no discernible effect on PMI.

### 3.2 Effects on semen quality at 48 h

**Figure 2:**
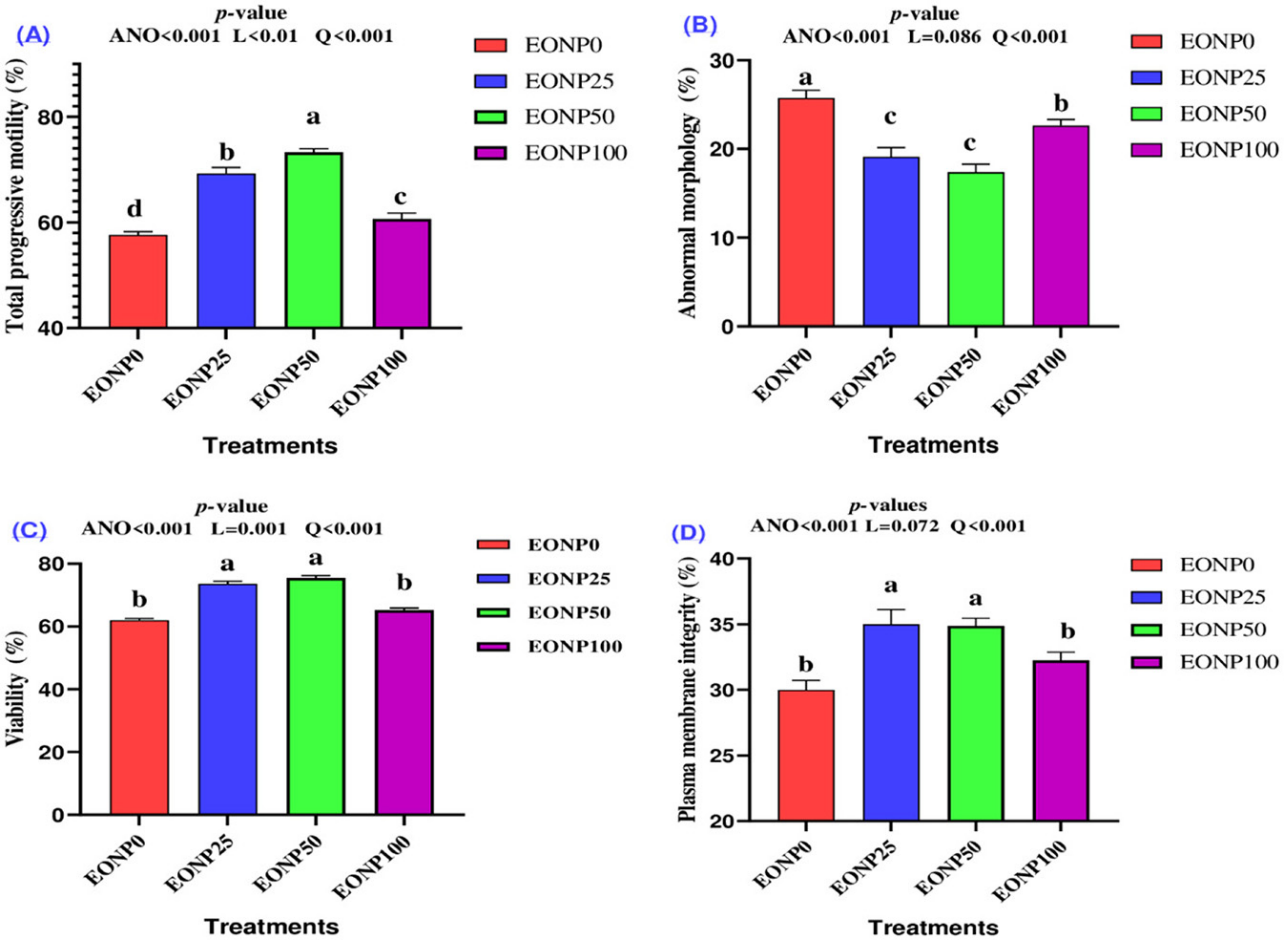
Outcomes of different concentrations of europium oxide nanoparticles (EONP; 0, 25, 50, and 100 ng/ml of extender**)** on chilled rabbit semen quality (4 °C for 48 h) designated as EONP0, EONP25, EONP50, and EONP100, respectively. Total progressive motility **(A)**, abnormal morphology **(B)**, viability **(C)**, and plasma membrane integrity **(D)**. Bars displaying discrete superscript letters (^a, b, c^) suggest statistically significant differences (*P* < 0.05) among the treatment groups.

Adding EONP to chilled rabbit extender significantly enhanced total progressive motility (Figure 2A) compared to the untreated group (*P* < 0.001), showing a quadratic dose-response relationship. The highest total progressive motility was noted in the EONP50 group (*P* < 0.001). EONP addition significantly decreased sperm abnormal morphology (Figure 2B), with the lowest values noted in the EONP25 and EONP50 groups compared to other groups (*P* < 0.01). A significant quadratic effect was noted for both viability (Figure 2C) and plasma membrane integrity (PMI) (Figure 2D), with EONP25 and EONP50 concentrations yielding the highest values (*P* < 0.001). These findings suggest that 25–50 ng/ml of EONP is the optimal level for improving rabbit semen quality during chilled storage.

### 3.3 Effects on semen quality at 72 h

**Figure 3:**
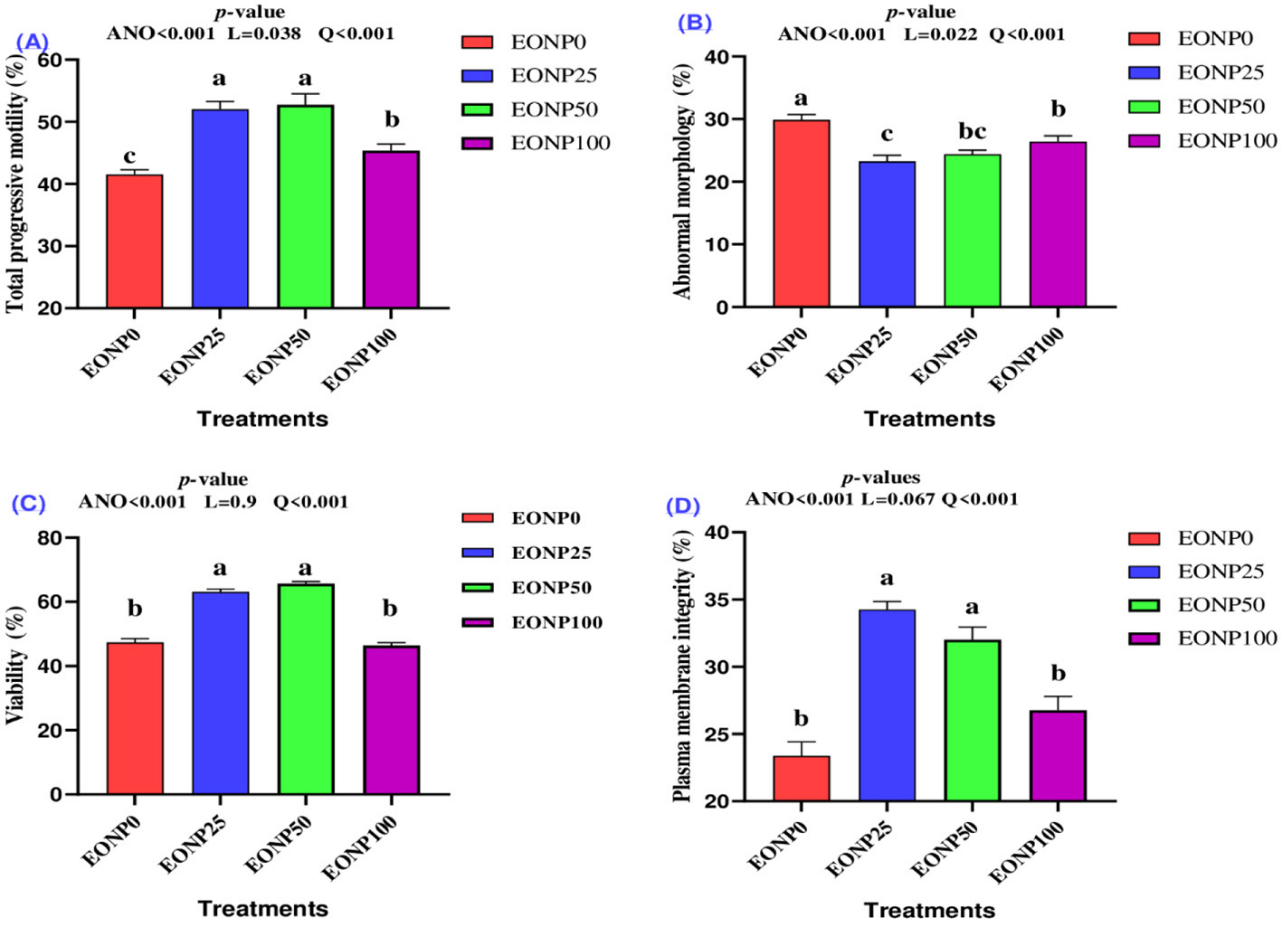
Outcomes of different concentrations of europium oxide nanoparticles (EONP; 0, 25, 50, and 100 ng/ml of extender) on chilled rabbit semen quality (4 °C for 72 h) designated as EONP0, EONP25, EONP50, and EONP100, respectively. Total progressive motility **(A)**, abnormal morphology **(B)**, viability **(C)**, and plasma membrane integrity **(D)**. Bars displaying discrete superscript letters (^a, b, c^) suggest statistically significant differences (*P* < 0.05) among the treatment groups.

Figure 3 illustrates the effects of varying EONP concentrations on rabbit semen during 72 h of chilled preservation at 4 °C. The supplementation of the extender with 25 or 50 ng/ml of EONP led to a quadratic increase in total progressive motility of chilled rabbit semen after 72 h of storage (Figure 3A). A quadratic effect was observed, where EONP at 25 or 50 ng/ml significantly reduced sperm abnormal morphology compared to the untreated control (EONP0) group (*P* < 0.05; Figure 3B). The EONP50 group showed similar results for sperm abnormalities compared to both the EONP100 and EONP25 groups (*P* > 0.05). Both sperm viability (Figure 3C) and plasma membrane integrity (PMI) (Figure 3D) exhibited a significant quadratic dose-response, with EONP25 and EONP50 concentrations proving most effective (*P* < 0.001). In contrast, the highest EONP concentration (100 ng/ml) did not statistically differ from the untreated control for either viability or PMI in rabbit semen chilled at 4 °C for 72 h.

### 3.4 Antioxidant competence

**Figure 4:**
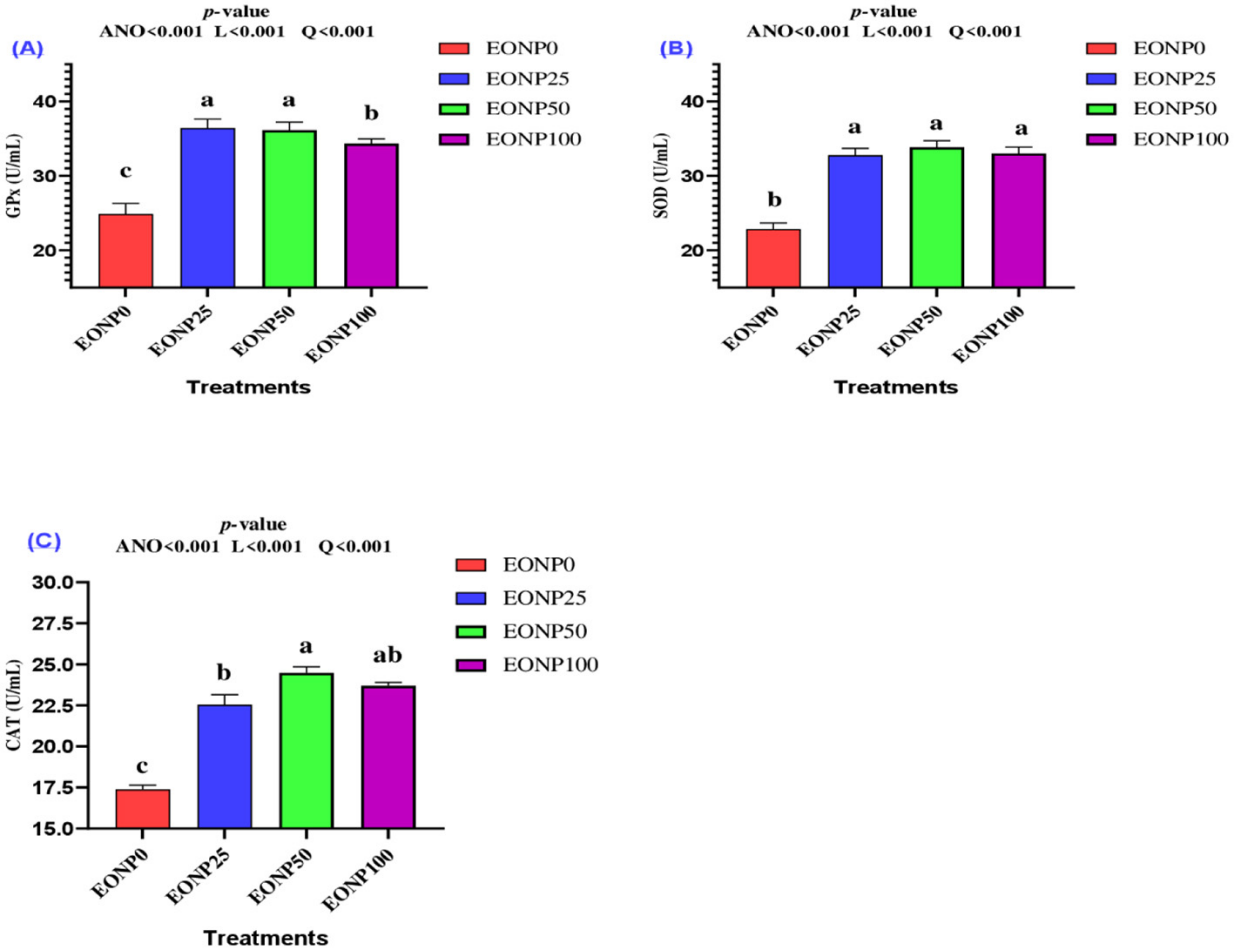
Outcomes of different concentrations of europium oxide nanoparticles (EONP; 0, 25, 50, and 100 ng/ml of extender**)** on antioxidant biomarkers (4 °C for 72 h) designated as EONP0, EONP25, EONP50, and EONP100, respectively. The antioxidant enzymes were GPx **(A)**, SOD **(B)**, and CAT **(C)**. Bars displaying discrete superscript letters (^a, b, c^) suggest statistically significant differences (*P* < 0.05) among the treatment groups.

Fortifying rabbit extenders with EONP resulted in a significant quadratic dose-dependent increase in GPx (Figure 4A), SOD (Figure 4B), and CAT (Figure 4C) levels compared to the EONP0 treatment (*P* < 0.01). All EONP treatment groups showed increased SOD levels compared to the control, indicating a significant quadratic effect (*P* < 0.001). The EONP25 and EONP50 groups exhibited the highest GPx levels (*P* < 0.001), with EONP50 being the most effective in enhancing CAT levels (*P* > 0.05).

### 3.5 Oxidative stress biomarkers

**Figure 5:**
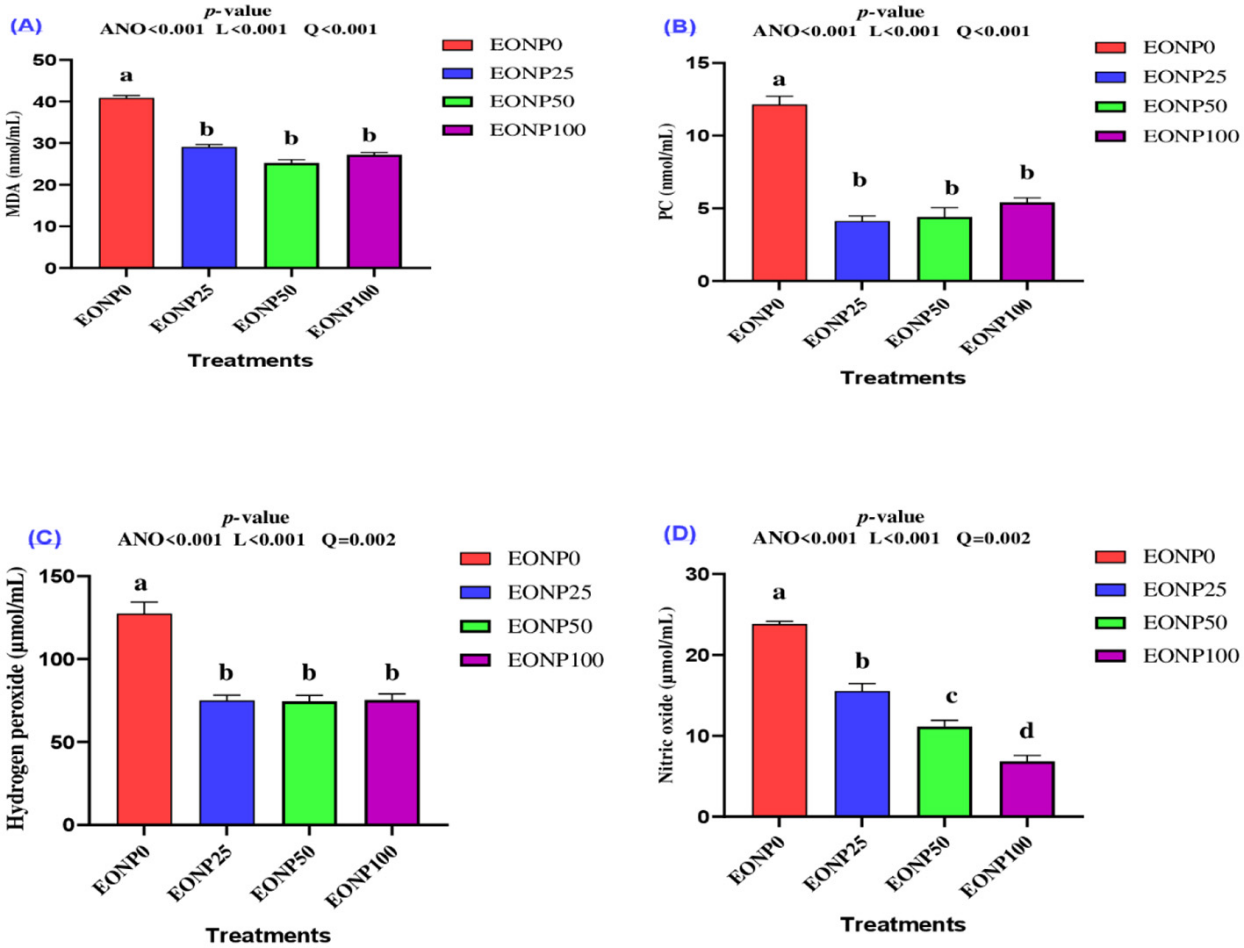
Outcomes of different concentrations of europium oxide nanoparticles (EONP; 0, 25, 50, and 100 ng/ml of extender**)** on oxidative stress biomarkers (4 °C for 72 h) designated as EONP0, EONP25, EONP50, and EONP100, respectively. The parameters assessed were malondialdehyde (MDA) **(A)**, protein carbonyl (PC) **(B)**, hydrogen peroxide (H_2_O_2_) **(C)**, and nitric oxide (NO) **(D)**. Bars displaying discrete superscript letters (^a, b, c^) suggest statistically significant differences (*P* < 0.05) among the treatment groups.

The inclusion of EONP in chilled rabbit semen resulted in a significant decrease in malondialdehyde (MDA) (Figure 5A), protein carbonyl (PC) (Figure 5B), hydrogen peroxide (H_2_O_2_) (Figure 5C), and nitric oxide (NO) (Figure 5D) levels compared to the control group (*P* < 0.001). All EONP groups showed significantly lower levels of oxidative markers, including MDA, PC, and H_2_O_2_, compared to the EONP-free extender (*P* < 0.001). Additionally, nitric oxide levels exhibited a gradual and significant dose-dependent decrease compared to the control group (*P* < 0.001). In conclusion, the addition of 25–100 ng/ml of EONP to chilled rabbit semen extenders resulted in a quadratic reduction in all measured oxidative stress markers (*P* < 0.001).

### 3.6 Mitochondrial bioenergetic enzymes

**Figure 6:**
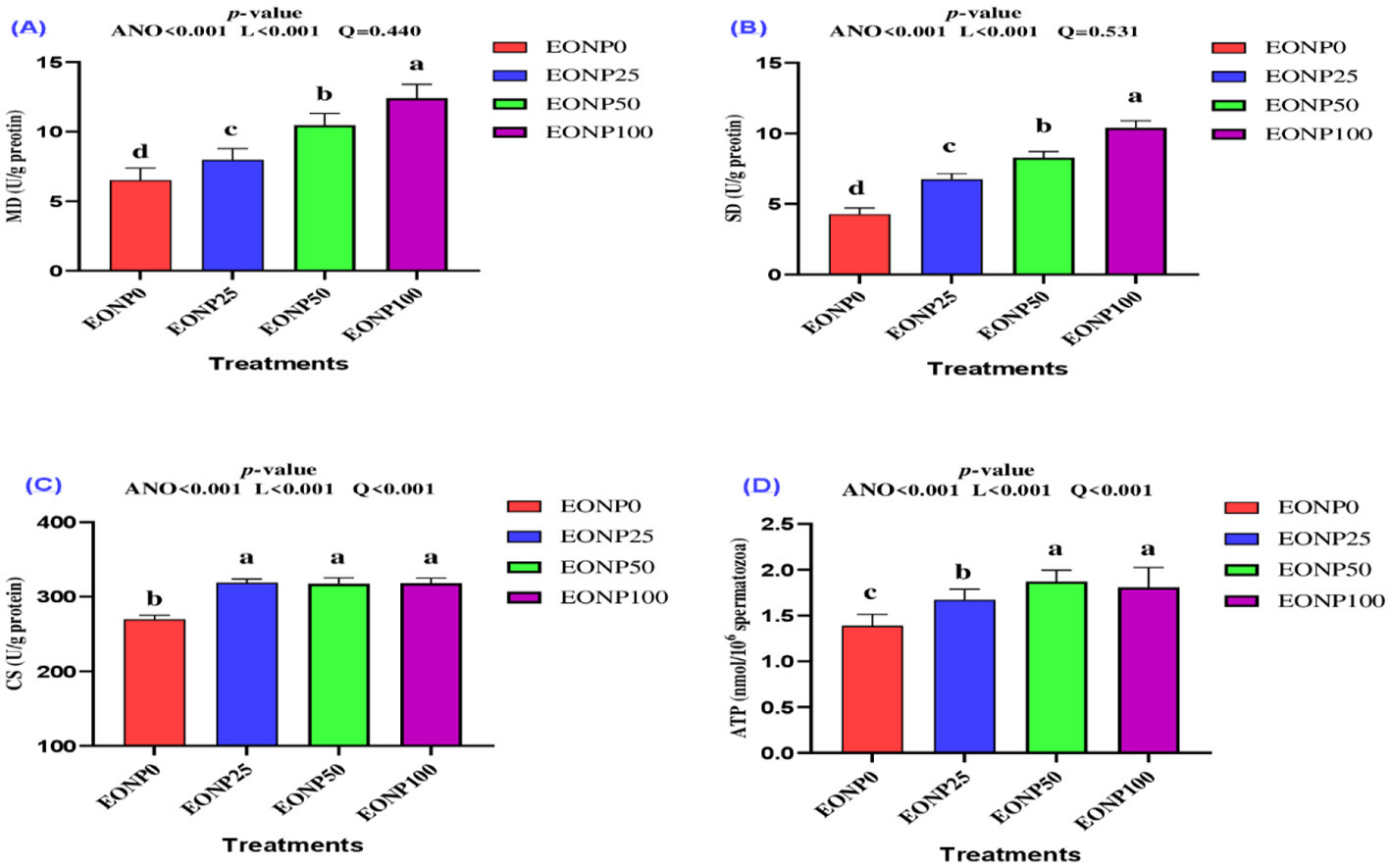
Outcomes of different concentrations of europium oxide nanoparticles (EONP; 0, 25, 50, and 100 ng/ml of extender**)** on mitochondrial enzymes of rabbit spermatozoa (4 °C for 72 h) designated as EONP0, EONP25, EONP50, and EONP100, respectively. Mitochondrial enzymes including MD **(A)**, SD **(B)**, citrate synthase (CS) **(C)**, and ATP **(D)**. Bars displaying discrete superscript letters (^a, b, c^) suggest statistically significant differences (*P* < 0.05) among the treatment groups.

Significant improvements in mitochondrial enzyme activities, including malate dehydrogenase (MD) (Figure 6A), succinate dehydrogenase (SD) (Figure 6B), and citrate synthase (CS) (Figure 6C), along with enhanced ATP levels (Figure 6D), were observed following EONP supplementation in chilled rabbit semen extenders. Adding 100 ng/ml of EONP resulted in the highest levels of MD and SD enzymes, showing a linear effect (*P* < 0.001). In terms of citrate synthase (CS), all EONP-supplemented extenders showed superior levels compared to the EONP-free extender. For ATP, while the EONP50 and EONP100 groups had the highest values, the EONP25 group notably had a quadratic effect on ATP levels relative to the control (*P* < 0.05). Overall, doses of 50 or 100 ng/ml of EONP were more effective in enhancing mitochondrial function than the 25 ng/ml concentration.

### 3.7 Principal component analysis (PCA)

**Figure 7:**
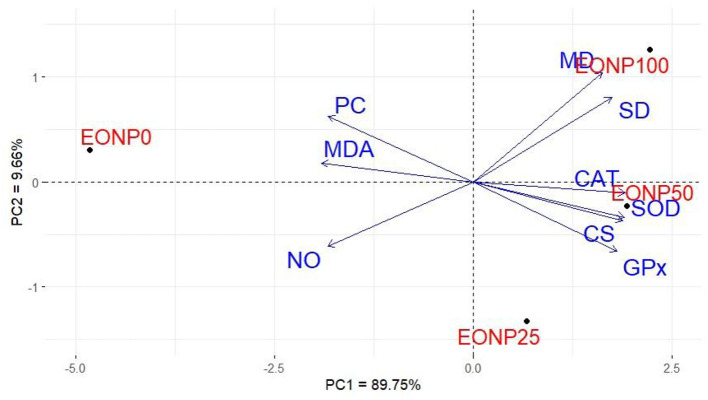
Principal component biplot for fortifying chilled rabbit semen with various levels of EONP (0, 25, 50, and 100 ng/ml) on sperm quality, antioxidant capacity, and mitochondrial enzymes after 72 h of preservation at 4 °C.

The complex interaction between different levels of EONP administration within the semen extender and the key parameters assessed in chilled rabbit semen was investigated using PCA (Figure 7). The parameters considered included sperm quality, oxidative/antioxidant markers, and mitochondrial enzyme activities. The first two PCAs explained the maximum variability of around 89.75% for PCA1 and 9.66% for PCA2 (Figure 7). PCA1 was correlated with EONP supplementation, antioxidant markers, mitochondrial enzyme activities, and several sperm quality parameters (progressive motility, viability, and plasma membrane integrity) on the positive side. Conversely, PCA2 showed an association between the different EONP concentrations (0, 25, 50, and 100 ng/ml) and oxidative markers and sperm abnormalities after 72 h of preservation at 4 °C on the negative side.

### 3.8 Correlation test using heatmap clustering

**Figure 8:**
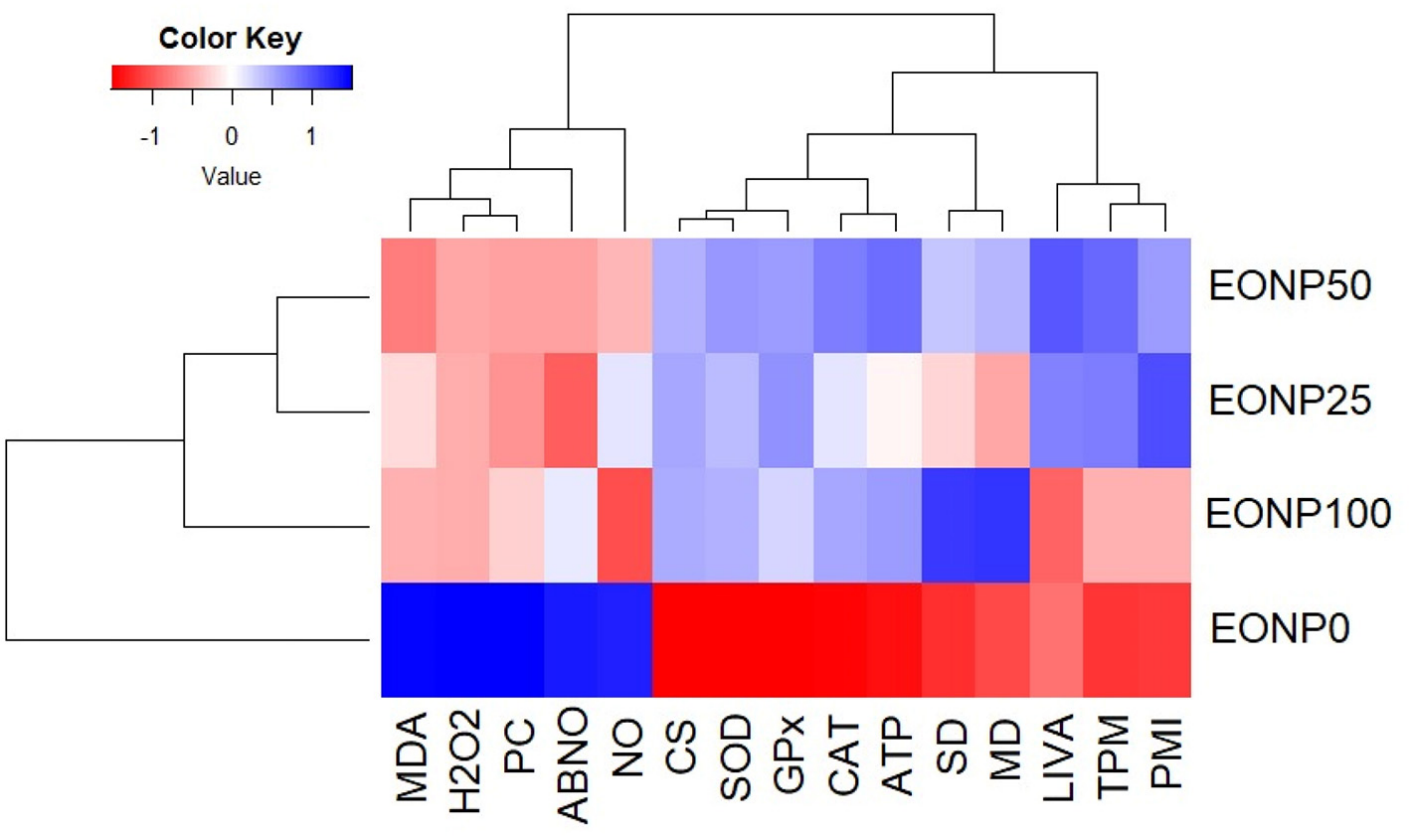
A heatmap and hierarchical clustering analysis were performed to evaluate the effects of fortifying chilled rabbit semen with various levels of EONP (0, 25, 50, and 100 ng/ml) on sperm quality, antioxidant capacity, and mitochondrial enzymes after 72 h of preservation at 4 °C.

We conducted heatmap clustering to analyze the physiological parameters, sperm quality, antioxidant markers, and mitochondrial enzyme activities in chilled rabbit semen, considering different EONP concentrations (0, 25, 50, and 100 ng/ml) in the extender. EONP addition was the primary factor distinguishing the major clusters (Figure 8). The control group (no EONP) consistently showed the lowest values (represented by red), while EONP supplementation generally led to the highest values for most parameters (represented by blue).

## 4 Discussion

Nanozymes are nanomaterials that exhibit enzyme-like catalytic activity. Researchers have identified numerous nanozymes that mimic the multifunctional roles of common natural enzymes such as GPx, CAT, and SOD. Our experiment demonstrates that supplementing chilled rabbit semen diluted with nanozymes like EONP significantly improves semen quality during 24, 48, and 72 h of storage at 4 °C. This improvement is evident in enhanced total progressive motility, sperm viability, and PMI, as well as reduced sperm abnormalities. Additionally, there is a notable increase in antioxidant enzyme activities (GPx, CAT, and SOD), enhanced mitochondrial bioenergetics activities (MD, SD, CS, ATP), and a decrease in oxidative stress indicators (MDA, PC, NO, and H_2_O_2_) after 72 h of storage at 4 °C.

Mitochondria are essential organelles in spermatozoa as they serve as the primary energy source for motility. However, they can also contribute significantly to oxidative stress (13). Therefore, it is crucial to maintain a delicate balance between reducing oxidative stress and enhancing energy production to ensure optimal sperm motility and improve fertilization outcomes.

Cooled rabbit semen is a common and effective system for artificial insemination (AI), often resulting in greater fertility rates than cryopreserved semen (1). Despite its advantages, ongoing research aims to further optimize the cooling process and reduce OS during 4 °C preservation. The goal is to enhance semen viability and ultimately improve fertility outcomes. Our findings demonstrate that nanozyme such as EONP, significantly improved sperm quality at concentrations of 25–ng/ml. Furthermore, at higher concentrations (50 or 100 ng/ml), EONP enhanced mitochondrial function by supporting mitochondrial enzymes, improving antioxidant competence, and reducing oxidative stress markers in sperm following 72 h of 4 °C preservation. These beneficial effects can be attributed to EONP's ability to mimic the catalytic activities of natural antioxidant enzymes. For instance, EONP is suggested to act like SOD, which catalyzes the dismutation of O^2·−^ into O^2^ and H_2_O_2_ (26, 27).

Europium is an inner transition metal that can also be used as a catalyst in certain chemical processes. Eu, which is a rare earth element with low toxicity and high biocompatibility, shows promising applications in biomedicine (21).

In human studies, cerium oxide nanoparticles significantly improved progressive motility and viability, while also reducing sperm abnormalities (28). Similarly, the addition of rare elements like cerium oxide nanoparticles has been shown to enhance sperm quality, including progressive motility, plasma membrane integrity, viability, and acrosome status in various livestock species (29, 30). This improvement is attributed to the anti-apoptotic properties of EONP, which seem to target the caspase-3 gene (31).

Cold storage can lead to increased oxidative stress, which can diminish the antioxidant capacity in the freezing medium. Therefore, the addition of antioxidants may enhance the overall antioxidant status and mitigate oxidative stress. In this study, it was observed that the inclusion of nanozymes, such as EONP (at a concentration of 50 ng/ml), significantly boosted antioxidant levels (GPx, CAT, and SOD) while reducing oxidative stress markers (MDA, PC, and H_2_O_2_). These results align with previous research that demonstrated the positive impact of antioxidants on chilled semen quality. For instance, a study on rabbit semen extender stored at 5 °C for 72 h showed that supplementation with glutathione and taurine notably improved sperm motility and viability (32). Lycopene provided significant protection during the storage of rabbit semen at 5 °C for two days (33). Mitochonic acid 5 notably improved semen quality (acrosomal integrity, motility, and PMI) in rams during storage at 4 °C. It achieved this by reducing oxidative stress and promoting ATP production and mitochondrial membrane potential (34). The improvements observed with EONP supplementation, particularly in sperm motility, viability, and PMI, are likely due to the antioxidant activity it provides to the chilled extender. This is supported by the increased levels of SOD, GPx, and TAC, which likely help reduce cold-induced oxidative stress and enhance sperm resilience. The concentration of benzoic acid in bull spermatozoa is positively correlated with fertility status, being notably higher in high-fertility bulls (8).

Cold shock significantly harms sperm by impairing membrane reorganization during capacitation, leading to decreased permeability and damage to acrosomal membranes (10, 34). This may explain the decline in acrosome integrity, plasma membrane, and sperm motility observed in our control group after 72 h at 4 °C. This study supports the findings of (30), who discovered that the addition of cerium oxide nanoparticles (25 and 50 μg/ml) to a freezing extender significantly improved the plasma membrane integrity, acrosome status, and viability of post-thawed chilled goat semen. In another study by Farag et al. (35), it was observed that cerium oxide nanoparticles (60 μg/ml) enhanced sperm membrane integrity, motility, and viability in chilled rabbit semen preserved at 4 °C for 72 h. The protective mechanism of EONP on sperm membranes is likely due to its ability to shield the sperm cell from damage caused by its high content of polyunsaturated fatty acids. By potentially replacing or mitigating injury to these cold-stressed fatty acids, EONP helps maintain crucial membrane structure and function.

Exposure of gametes, particularly sperm, to cold stress may impair their antioxidant defense mechanisms and induce oxidative stress (1, 2). Semen samples without EONP showed reduced GPx, CAT, and SOD activity in this study. In contrast, semen samples supplemented with EONP at different concentrations exhibited higher levels of these enzymes, indicating the antioxidant effect of EONP. Additionally, oxidative markers such as PC, MDA, NO, and H_2_O_2_ were reduced, especially with high doses of EONP (100 ng/ml), suggesting EONP's ability to scavenge oxidative stress in semen samples exposed to cold stress. These results are consistent with previous findings by Machhi et al. (36), who demonstrated that Europium-doped cerium oxide nanoparticles exhibit anti-oxidative action by reducing ROS, PC, and MDA levels in Alzheimer's disease. The anticancer and antimicrobial activities of EONPs may also be attributed to their ability to scavenge oxidative stress (37).

Several studies have confirmed that adding metallic nanoparticles, especially those containing rare elements, can improve the antioxidant status and reduce oxidative stress in preserved semen (28, 38). Supplementing goat freezing media with 75 μg/ml of cerium oxide nanoparticles significantly enhanced PMI, viability, and acrosome integrity. This improvement occurred by bolstering antioxidant enzymes such as SOD and CAT (30). This imbalance occurs because cold exposure increases ROS production while simultaneously reducing the activity of crucial antioxidant enzymes (1). Incorporating metallic nanoparticles into semen extenders may boost sperm's resistance to cold stress during preservation (39). This could be due to the unique chemical and physical actions of these nanozymes. In our study, we examined substantial developments in antioxidant indices and decreases in OS indicators in chilled rabbit semen, especially when using EONP at concentrations of 50 or 100 ng/ml. According to some authors, EONP's antimicrobial activity could explain this function (31).

The crucial role of mitochondria, cellular powerhouses, in sperm function has a significant impact on fertility outcomes in rabbits (23). This study demonstrates that the addition of EONP significantly enhanced various indicators of sperm mitochondrial health, such as the activity of enzymes like CS, MD, and SD, as well as overall ATP levels. These findings strongly suggest that EONP helps maintain the essential structure of sperm mitochondria, thereby contributing to their prolonged function. These results are consistent with those of Huang et al. (40) who highlighted the vital role of Eu ions in enhancing mitochondrial function and biosynthesis in bone mesenchymal stem cells (41). It is possible that EONP is responsible for this improvement, potentially by regulating energy production in sperm mitochondria, which subsequently impacts sperm physiology.

High activity in mitochondrial enzymes such as MD, CS, and SD reflects an accelerated TCA cycle, confirming that the sperm cells are efficiently producing ATP (12, 13, 17). Studies have shown that adding oleic or palmitic acid to boar semen freezing media significantly boosts both MD and SD activities (13), accompanied by a substantial enhancement in ATP synthesis. Citrate synthase (CS) is a crucial enzyme in cellular energy production, particularly a key indicator of mitochondrial health and activity. Targeting mitochondrial enzymes may improve sperm function, enhancing the sperm's ovum-penetrating ability and increasing fertilization capacity (11).

In our study, chilled rabbit semen supplemented with EONP showed higher levels of MD, CS and SD, indicating improved metabolic activity. We also observed a significant reduction in harmful byproducts like MDA, H_2_O_2_, PC, and nitric oxide, suggesting a decrease in oxidative stress. Previous studies have suggested that this improvement in mitochondrial function was due to the antimicrobial activity of EONP (20). It has been demonstrated that Eu^3+^ enhances the energy-dependent K^+^ transport into the matrix in *Vertebrate Myocardium* (42). Similar improvements have also been observed in ram semen, with studies reporting comparable results using mitochonic acid 5 (34), and β-Nicotinamide mononucleotide (43). This suggests that the active compounds in EONP likely work in two ways: first, by promoting beta-oxidation, which boosts the levels of mitochondrial enzymes critical for energy production, and second, by scavenging the oxidative stress triggered by the cooling process. EONP plays a key role in anabolic pathways essential for sperm's energy generation and the creation of critical constituents, such as phospholipids. These contributions are fundamental to sperm motility, viability, and overall functional integrity. Supporting these observations, Abdelnour et al. (10) reported that L-carnitine supplementation significantly enhanced mitochondrial function in cryopreserved rabbit semen. Given the constraints of this study's resources, we evaluated some oxidative markers, mitochondrial bioenergetic enzymes and antioxidant enzymes exclusively at the experimental endpoint. To gain a deeper understanding of the damaging effects of short-term semen storage, future investigations should employ metabolomics and proteomics assays. This expanded knowledge is vital for creating new protocols that will bolster the sustainability and practical application of AI using chilled semen.

## 5 Conclusion

Rabbit sperm quality is negatively affected by low temperatures, leading to compromised mitochondrial function, heightened oxidative stress, and reduced antioxidant capacity. Our findings show that adding 25 or 50 ng/ml of EONPs to the semen extender significantly improved sperm motility and plasma membrane integrity in chilled rabbit semen. Notably, the doses of 50 and 100 ng/ml of EONP also decreased oxidative markers, enhanced antioxidant enzyme activity, and improved sperm mitochondrial bioenergetics. In summary, our results suggest that europium nanoparticles can be effectively utilized to preserve rabbit sperm quality during hypothermic storage at 4 °C. Subsequent investigations are necessary to determine the optimal dosage and fully elucidate their effects on underlying molecular pathways.

## Data Availability

The original contributions presented in the study are included in the article/Supplementary material, further inquiries can be directed to the corresponding author/s.
