# Supplementary material for: Europium oxide nanoparticles (EONP) enhance the cryoprotective effects of chilled rabbit semen preservation via antioxidant and mitochondrial enhancement
Source: Front Vet Sci. 2026 Jan 27;12:1753818. doi: 10.3389/fvets.2025.1753818 (PMC12886018; doi:10.3389/fvets.2025.1753818)
Supplement: Supplementary file 1 [file Image_1.pdf]

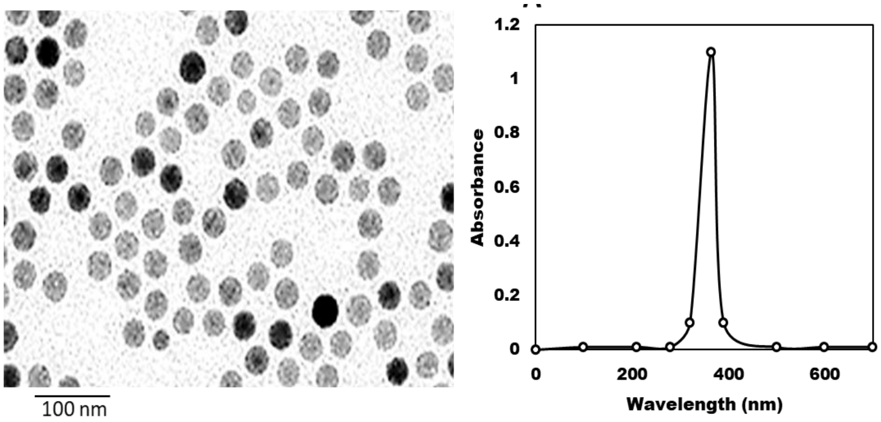

Figure S1. Characterization of europium oxide nanoparticles (EONP): A. The UV wavelength of EONP is 363nm. B. TEM analysis shows that the size of EONP ranges from 37-92nm. C. The average size of EONP is 46nm. D.
